# ReCount: A multi-experiment resource of analysis-ready RNA-seq gene count datasets

**DOI:** 10.1186/1471-2105-12-449

**Published:** 2011-11-16

**Authors:** Alyssa C Frazee, Ben Langmead, Jeffrey T Leek

**Affiliations:** 1 Department of Biostatistics, The Johns Hopkins University Bloomberg School of Public Health, 615 North Wolfe Street, Baltimore, MD 21205, USA

## Abstract

**1 Background:**

RNA sequencing is a flexible and powerful new approach for measuring gene, exon, or isoform expression. To maximize the utility of RNA sequencing data, new statistical methods are needed for clustering, differential expression, and other analyses. A major barrier to the development of new statistical methods is the lack of RNA sequencing datasets that can be easily obtained and analyzed in common statistical software packages such as R. To speed up the development process, we have created a resource of analysis-ready RNA-sequencing datasets.

**2 Description:**

ReCount is an online resource of RNA-seq gene count tables and auxilliary data. Tables were built from raw RNA sequencing data from 18 different published studies comprising 475 samples and over 8 billion reads. Using the Myrna package, reads were aligned, overlapped with gene models and tabulated into gene-by-sample count tables that are ready for statistical analysis. Count tables and phenotype data were combined into Bioconductor ExpressionSet objects for ease of analysis. ReCount also contains the Myrna manifest files and R source code used to process the samples, allowing statistical and computational scientists to consider alternative parameter values.

**3 Conclusions:**

By combining datasets from many studies and providing data that has already been processed from. fastq format into ready-to-use. RData and. txt files, ReCount facilitates analysis and methods development for RNA-seq count data. We anticipate that ReCount will also be useful for investigators who wish to consider cross-study comparisons and alternative normalization strategies for RNA-seq.

## Background

RNA-seq, or short-read sequencing of mRNA, has emerged as a powerful and flexible tool for studying gene expression [1]. As with other new technologies, the analysis of RNA-seq data requires the development of new statistical methods. Data from many RNA-seq experiments are publicly available, but processing raw data into a form suitable for statistical analysis remains challenging [2]. This difficulty together with the high cost of using second-generation sequencing technology means that most computational scientists have only a limited number of samples to work with [3]. However, replication is critical to understanding biological variation in RNA-sequencing [4].

The Gene Expression Omnibus [5] is a useful repository that contains both processed and raw microarray data, but there is no comparable resource for processed RNA-seq data. We have compiled a resource, called ReCount, consisting of aligned, preprocessed RNA-seq data from 475 samples in 18 different experiments. Our database makes it easier for statistical and bioinformatics researchers to analyze RNA-seq count data using standard tools such as R, Bioconductor [6], and MATLAB. The aligned and preprocessed data in ReCount can be directly analyzed, used to develop and compare new methods for analysis, or examined to identify cross-study effects. The ReCount database also contains the Myrna manifest files and R source code used to process the samples, allowing statistical and computational scientists to consider alternative parameter values.

## Construction and Content

### Content

We collected data from the 18 experiments described in Table 1[7-24]. For each experiment, ReCount contains a. txt-format count table encoding, for each sample, the number of reads overlapping each gene included in the Ensembl [25] annotation of the given organism's genome. ReCount also includes manually curated phenotype information (e.g. sex, strain, time point) for each sample, available as a. txt file. Count and phenotype tables were compiled into ExpressionSet objects, which are downloadable from ReCount and can be easily loaded and analyzed using standard Bioconductor tools in R.

**Table 1:** Datasets available for download (truncated to 35 bp)

Study	Organism	Number of bio reps	Number of reads
BodyMap	human	19	2,197,622,796
Cheung	human	41	834,584,950
Core	human	2	8,670,342
Gilad	human	6	41,356,738
MAQC	human	14	71,970,164
Montgomery	human	60	*886,468,054
Pickrell	human	69	*886,468,054
Sultan	human	4	6,573,643
Wang	human	22	223,929,919
Katz	mouse	4	14,368,471
Mortazavi	mouse	3	61,732,881
Trapnell	mouse	4	111,376,152
Yang	mouse	1	27,883,862
Bottomly	mouse	21	343,445,340
Nagalakshmi	yeast	4	7,688,602
Hammer	rat	8	158,178,477
modENCODE - worm	worm	46	1,451,119,823
modENCODE - fly	fly	147	2,278,788,557

The "Number of bio reps" column contains the number of individual samples contained in the dataset, while the "Number of reads" column displays the number of uniquely aligned reads that were used to create the count table. A version of this table and an analogous table for the downloadables created by removing Myrna's truncate option are available on the website.

### Construction

To construct count tables, we used the Amazon Elastic MapReduce version of Myrna 1.1.2 [26]. As input, Myrna requires a manifest file listing URL locations for all sequencing read files for each sample. Myrna manifest files are available as part of ReCount; most URLs in these files refer to reads stored in the NCBI Sequence Read Archive (SRA) [27].

For studies consisting of paired-end sequencing data, only the first mate of each pair was considered. Many studies also included technical replicates, which were processed using Myrna's pool-tech-reps option. This option pools the reads from technical replicates prior to alignment and analysis. Other options passed to Myrna were bowtie-args = "-v 2 -m 1", gene-footprint = intersect, and from-middle. The gene-footprint = intersect parameter causes a "union intersection" gene model to be used. The bowtie-args parameters specify that no more than two mismatches are allowed for a read alignment to be valid and that reads with multiple alignments are discarded. The from-middle argument designates that the number of bases considered when overlapping a read's alignment with a gene footprint should be measured from the middle of the read (rather than the 3' or 5' end). Finally, we provide count tables and ExpressionSets created using Myrna's truncate = 35 option, which truncates reads longer than 35 bp to 35 bp. For using data from multiple studies at once, the truncation makes studies more comparable to each other; it also decreases the likelihood that a read will span a splice junction and therefore be discarded. However, for researchers who wish to utilize the full read length, we also provide count tables and ExpressionSets created without the truncate option.

Count tables presented in ReCount have not yet been normalized. During analysis, gene counts in each sample are commonly normalized by dividing by the 75th percentile of the distribution of non-zero gene counts in the sample, as suggested previously [11], but the data presented in ReCount allows researchers to develop, evaluate, and compare alternative normalization schemes.

## Utility and Discussion

### User Interface

The ReCount website features an interactive version of Table 1. ExpressionSets, count tables, and phenotype tables are downloadable from the table. Manifest files used with Myrna, specific Myrna commands used, and R code used to create ExpressionSets are also available for download at the ReCount website. These scripts allow researchers to compare the effects of alternative normalization or alternative Myrna parameterization to the canonical versions of the datasets contained in the ReCount database. The site also contains further details about the contents of the downloadables as well as a set of R commands that may be useful when working with ExpressionSets.

### Utility: Example Applications

ReCount facilitates studies that are not possible using only a small number of samples from a single study. Here we present two toy examples that illustrate the potential utility of the ReCount database. Both examples used datasets created with reads truncated to 35 bp.

#### Application 1: Comparison of normalization methods

Count tables presented in ReCount have not yet been normalized, which facilitates comparisons between normalization and preprocessing approaches. As an example, we compared 75th percentile normalization [11] with quantile normalization [28] using data collected on two different strains of mice (the bottomly dataset available in ReCount [20]). We analyzed 36,536 total genes, first removing genes with zero counts or that showed no variation across samples (23,697 genes). For both types of normalization, each gene was tested for differential expression between the two strains using an F-test. There were 696 genes that were differentially expressed at a false discovery rate of 5% (a Benjamini-Hochberg correction for multiple testing [29] was used) in both analyses, while 177 were only differentially expressed using quantile normalization and 35 were only differentially expressed using 75th percentile normalization. The set of differentially expressed genes for the quantile normalization scheme is a bit larger than the set for the 75th percentile normalization, but the overlap is still quite large. This simple analysis demonstrates a method for comparing normalization schemes; it also illustrates that results of a differential expression analysis differ very little based on which of these two well-established normalization schemes was used.

#### Application 2: Analysis using data from multiple studies

**Figure 1:**
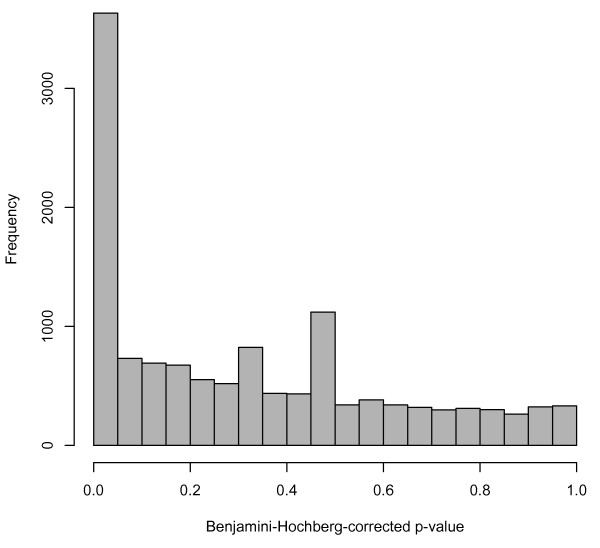
**Histogram of adjusted p-values from differential expression analysis on the 29 samples included in both Cheung and Montgomery**. The p-values in the histogram are from paired t-tests on the 25% of genes with nonzero counts in at least one of the two studies. The peak near zero is somewhat indicative of technical variability between the two studies.

Availability of comparable data from many studies facilitates analyses that previously may have been quite cumbersome. As an example, we consider the Cheung [8] and Montgomery [12] data. These two studies assayed 29 of the same individuals. The Cheung group sequenced immortalized B cells, and Montgomery et. al. used lymphoblastoid cell lines, so the types of cells used in sequencing were very similar. Therefore, examining these 29 samples and comparing gene expression between the two studies could provide insight into some of the technical variability present in RNA-seq. As a very basic analysis of differential expression, we compared subjects' measured gene expression in the Cheung study vs. the Montgomery study using a parametric paired t-test on each gene in the table. Genes for which the difference in gene expression was significantly different from zero were considered differentially expressed between studies. (A Benjamini-Hochberg correction for multiple testing was performed; a false-discovery-rate cutoff of 0.05 was used to determine significance). Of the 52,580 genes tested, 3,633 (6.9%) were found to be differentially expressed between the studies. We also note that 39,752 genes (75.6%) could not be tested for differential expression because all counts were zero in both samples. So, of the 12,828 genes that had nonzero gene counts for at least one sample in one of the studies, 3,633 (28.3%) were differentially expressed. This pattern can be seen in the histogram of the adjusted p-values (Figure 1). This analysis shows evidence of batch-like effects in RNA-seq; differential expression would ideally be quite rare since the same people and similar cell types were analyzed in each study.

**Figure 2:**
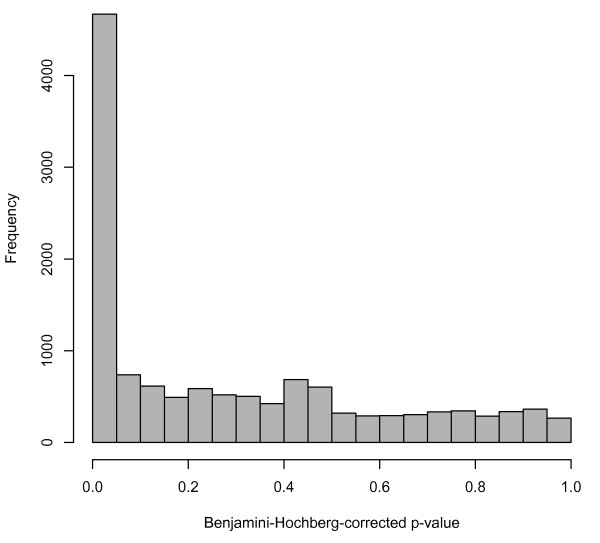
**Histogram of adjusted p-values from analysis of differential expression between YRI and CEU populations**. The p-values in the histogram are from two-sample t-tests on the 25% of genes with nonzero counts in at least one of the two studies. The peak near zero indicates differential gene expression that may result from either technical or biological variability.

As another example of an analysis using multiple datasets found in ReCount, we performed a simple differential expression analysis on the Montgomery data [12] and the Pickrell data [13], which is a proxy for an analysis of differential expression between ethnicities: the Montgomery group sequenced Utah residents with northern- or western-European ancestry (the HapMap CEU population), and the Pickrell group sequenced Yoruba people in Ibadan, Nigeria (the HapMap YRI population). Previous research has addressed this question (e.g. [30,31]), but ReCount facilitates investigation of alternative approaches to the problem. As a starting point, we performed an analysis similar to the previous one: for each of 52,580 genes, we performed a parametric two-sample t-test on the 75th-percentile normalized counts from the Montgomery and Pickrell data, using a Benjamini-Hochberg correction for multiple testing and considering a gene differentially expressed between CEU and YRI populations if its Benjamini-Hochberg-corrected p-value was less than 0.05. Of the 52,580 genes tested, 4,669 (8.9%) were found to be differentially expressed. These 4,669 genes comprise 36.0% of the genes tested for differential expression, i.e., genes with nonzero counts in at least one of the populations (Figure 2). We notice that these percentages are slightly higher than the percentages reported in the previous analysis, which is unsurprising, since both technical and biological variability are present here, whereas the variability in the previous analysis was mostly technical. Follow-up analysis could be performed for these 4,669 genes of interest, e.g., this set could be compared with sets of differentially expressed genes found in previous studies, or expression patterns in individual genes could be visualized. We present these basic analyses as starting points for researchers wishing to simultaneously utilize multiple datasets from ReCount.

## Discussion

ReCount's preproccessed, freely-available data compatible with common statistical software will encourage statisticians interested in methods development to tackle problems arising in RNA-seq data analysis. By providing a large amount of RNA-seq data in a central, accessible location, ReCount facilitates analyses like those above and several others. For example, another interesting application may be to attempt to replicate findings from other studies (e.g., percentage of differentially expressed genes). Additionally, all commands and manifest files used in preprocessing are available on the website, so users can create their own count tables should they desire alternative parameterizations: e.g., alternative alignment parameters can be passed to Bowtie, the truncation length can be changed, or the pool-tech-reps option can be removed.

## Conclusions

ReCount addresses two key issues for statistical researchers interested in RNA sequencing: (1) small sample sizes in many available studies and (2) computational difficulties in developing analysis-ready RNA-sequencing data. By providing Myrna manifest files and R scripts that reproduce the count tables in ReCount, our database also allows for flexible exploration of a large number of organized RNA-sequencing datasets. We anticipate that ReCount will be useful to both the statistical and bioinformatics community as a resource for readily analyzable RNA-sequencing data.

## Availability and Requirements

ReCount is publicly accessible at http://bowtie-bio.sf.net/recount.

## Competing interests

The authors declare that they have no competing interests.

## Authors' contributions

JL and BL conceived the study. AF and BL contributed new methods and built the database. AF, JL and BL wrote the paper. All authors read and approved the final manuscript.
